# Relationship between Thyroid Status during the First Trimester of Pregnancy and Neonatal Well-Being

**DOI:** 10.3390/nu13030872

**Published:** 2021-03-07

**Authors:** Maria Teresa Murillo-Llorente, Francisco Llorca-Colomer, Marcelino Pérez-Bermejo

**Affiliations:** 1 SONEV Research Group, School of Medicine and Health Sciences, Catholic University of Valencia San Vicente Mártir, C/Quevedo n° 2, 46001 Valencia, Spain; mt.murillo@ucv.es; 2 School of Medicine and Health Sciences, Catholic University of Valencia San Vicente Mártir, C/Quevedo n° 2, 46001 Valencia, Spain; llorfran@mail.ucv.es

**Keywords:** iodine supplements, iodized salt, gestation, APGAR score, newborn, fetal well-being

## Abstract

Iodine is an essential micronutrient for the synthesis of thyroid hormones. The proper functioning of the thyroid axis is essential for the normal development of the nervous system, especially in the first trimester of gestation. The aim of the present study was to analyze the perinatal outcomes, anthropometry, and APGAR test scores of newborns and to relate them to maternal thyroid status. A total of 190 newborns participated in the study. No correlation was found between thyroid stimulating hormone (TSH) and maternal ioduria values in the first trimester of gestation with the weight or length of the newborn, or the APGAR score at one minute after birth. However, we found significant differences between the APGAR scores of children whose mothers had an iodine sufficiency level in the first trimester compared to the children of mothers with iodine deficiency. Similarly, the APGAR scores of children whose mothers had a TSH > 4 have significantly better APGAR scores than the children of mothers with a TSH < 4. Likewise, we found significant differences between the measurements of the newborns depending on whether their mothers smoked. The children of mothers who took iodine supplements or iodized salt obtained the highest APGAR score at one and five minutes after birth. It is essential to focus on recommending adequate consumption of iodine supplements and iodized salt prior to gestation and at least during the first trimester to achieve better fetal well-being.

## 1. Introduction

The stress the thyroid gland undergoes generated by the considerable anatomical and functional repercussions of pregnancy can cause hypothyroidism in women who have an impaired thyroid reserve or iodine deficiency [1]. Thyroid hormones are in charge of regulating the metabolism and the way it uses energy, thus affecting the growth and development of all organs, especially the brain [2].

Iodine is an essential micronutrient for the correct function and synthesis of thyroid hormones and, therefore, any degree of iodine deficiency (mild, moderate, or severe) during pregnancy and lactation can affect the thyroid function of both the mother and the fetus [3]. The proper functioning of the thyroid axis is essential for the normal development of the nervous system, especially in the first trimester of gestation when fetal thyroid hormone concentration depends directly on the maternal hormonal contribution. Pregnant women with hypothyroidism are at a higher risk of suffering from preeclampsia, placental abruption, and postpartum hemorrhage, among others [3,4,5,6,7].

Iodine is a trace element of vital importance that cannot be synthesized or stored by the human body. Therefore, minimum requirements for an optimal state of health are not guaranteed by just diet; consequently, foods enriched with iodine must be added [7]. Although iodine is widely distributed in nature, most of it is found in ocean water, where it can reach a concentration of 50 µg/L. The surface of the earth is poor in iodine, especially in inland areas, far from marine waters [8].

According to the World Health Organization (WHO), iodine deficiency is the leading cause of mental retardation and cerebral palsy worldwide [9]. It is estimated that one-third of the world’s population lives in iodine-deficient areas [2,10]. After reviewing studies carried out in Spain, the WHO issued a report in 2007 in which it ranks this nation among the iodine-sufficient countries [11,12]. However, these figures show variations in the pregnant population, in which deficits in iodine figures under 150 μg/L can be observed in regions such as Castilla-La Mancha, the Basque Country, Catalonia, Madrid, Castile and Leon, Andalusia and the Valencian Community [12,13,14].

A global public health priority is the eradication of iodine deficiency in view of its negative consequences on brain and psychomotor development in the early stages of life [15,16]. The best strategy to avoid iodine deficiency in most of the population is the regular consumption of iodized salt, as recommended by the WHO, the United Nations International Children’s Emergency Fund (UNICEF), and the International Council for Control of Iodine Deficiency Disorders (ICCIDD) [17]. Different organizations recommend providing pregnant women with an extra supplement of between 200–300 μg of iodine/day [18,19].

There is a general consensus on the importance of treating hypothyroidism early in pregnancy to prevent complications [20]. Despite the fact that a relationship has been confirmed between subclinical hypothyroidism and hypothyroxinemia with obstetric difficulties and fetal psychomotor and neurocognitive impairment [21,22,23,24,25,26], there is still no clear evidence of the positive effects of thyroxine treatment in these cases [27,28].

During the first trimester of gestation, thyroid stimulating hormone (TSH) levels are partially suppressed due to the stimulatory effect of human chorionic gonadotrophin (hCG) on the thyroid gland. This effect can be modulated by different factors such as thyroid autoimmunity, body mass index, age, parity or smoking habit [29,30,31]. Different studies [32,33,34,35] have reported a causal relationship between maternal smoking and low birth weight, as well as a reduction in length and other anthropometric measures, causing an overall effect on fetal body composition. However, they found no significant differences between APGAR score values for the children of smoking mothers and non-smoking mothers.

For these reasons, the main objective of our work was to analyze the perinatal outcomes of newborns, type of delivery, anthropometry, and APGAR test scores and to relate them to maternal TSH levels and iodine intake during the first trimester of gestation.

## 2. Materials and Methods

We carried out an observational, descriptive, and longitudinal study in the Health Department of La Ribera (Valencia). The study was approved by the Ethics and Clinical Research Committee of La Ribera University Hospital (accepted on 13 December 2013), and written informed consent was obtained from all participants. This study complies with the principles laid down in the Declaration of Helsinki.

The pregnant women were selected consecutively, and the collection of samples was carried out consecutively by a team of 18 trained midwives. Pregnant women in the first trimester of pregnancy, who were residents in the study area, were included in the study. They were healthy and over 16 years old and presented no thyroid pathology at the time of inclusion.

Pregnant women undergoing treatment with drugs that influence and interfere with iodine metabolism (heparin, glucocorticoids, β-adrenergic blockers) and women who underwent thyroid hormone analysis in a laboratory other than that of the University Hospital of La Ribera were excluded. 

After taking data from pregnant women in the first trimester of pregnancy, a follow-up was carried out to be able to analyze the newborns.

Our study comprised a sample of 190 newborns (whose mothers completed a data collection form, in addition to having their thyroid function analyzed in the first trimester of gestation) and subsequently various neonatal physiological, biochemical, and anthropometric variables were collected in the same hospital during the years 2015 and 2016.

### 2.1. Laboratory Methods

All pregnant women provided an aliquot of non-first void urine sample (first-void samples may underestimate iodine levels [36]) collected in a sterile plastic bottle for determinations of urinary iodine. All urine samples were carried out from 08:00 a.m. to 10:00 a.m. and stored at −20 °C until analysis in the iodine laboratory. They also provided fasting peripheral venous blood samples from an antecubital vein early in the morning. Samples were centrifuged and serum was stored at −40 °C until the analysis. All blood samples were taken at the first antenatal visit (gestation weeks 5–13). Thyroid-stimulating hormone (TSH) determination was performed by the TSH3-Ultra ADVIA Centaur assay and we divided the mothers into 2 groups, depending on whether their TSH value was above or below 4 mIU/L [18]. Values are expressed as mUI/L for TSH. Ioduria was determined using the Dunn colorimetric technique [37], and a desirable value of >150 μg/L in pregnancy was established [18].

### 2.2. Variables

The variables included in the study were: socio-demographic (maternal age), obstetric (gestational age, type of delivery, delivery complications), anthropometric (newborn length and weight), iodine supplementation, iodized salt intake, smoking habit, and analytical variables (TSH and ioduria before week 12 of gestation).

### 2.3. Data Analysis

Data were entered and stored in an MS Excel file and then transferred to SPSS v.23 software (SPSS Inc., Chicago, IL, USA) for statistical analysis. Normality of the data distribution was determined using the Kolmogorov–Smirnov test. Outliers were detected using the Reed criterion [38]. The same criterion applies for minimum values. The data were presented using the central Confidence Interval (CI) of 95% (percentile 2.5 and 97.5) for TSH, mean and standard deviation (SD) in the case of normal distribution or median, and interquartile range (IQR) if this was not the case. For the analysis of continuous variables, the comparison between the values was made by unpaired Student’s *t*-test in the case of normality. The nonparametric Mann–Whitney test was used when the normality hypothesis was rejected when comparing two samples. The relationship between continuous variables was established using Pearson’s correlation coefficient or Spearman’s non-parametric correlation coefficient as needed. Two-sided *p* < 0.05 was considered statistically significant.

## 3. Results

All deliveries occurred after week 35 of gestation, the majority (87.4%) between weeks 38 and 41. Of them, 8.5% occurred between week 35 and 37 and 4.1% in week 42. Table 1 describes the sociodemographic characteristics of the sample.

There are statistically significant differences between weight and length according to sex (*p* < 0.05). On average, boys weigh 162.03 g more than girls (95% CI 41.17–282.89). Similarly, boys are 1.03 cm longer than girls (95% CI 0.44–1.62).

Of the 127 natural births, none had complications. Most complications occurred in instrumented deliveries (90.4%) and Caesarean sections (71.4%).

Table 2 describes the clinical characteristics of the sample. The maternal values correspond to the mothers of the 190 children analyzed and not to the total of 261 women participating in the previous study.

There were statistically significant differences in ioduria levels in the first trimester of pregnancy, taking into account the minimum level of ioduria of 150 μg/L recommended by the World Health Organization (WHO) [18]. Similarly, there were significant differences in the level of TSH, considering a cut-off point of 4 mIU/L [18].

We found statistically significant differences (*p* < 0.05) between ioduria values of those that consumed iodine supplements in first trimester of pregnancy (50.2 μg/L, 109.7–167.65) and those who did not consume it (49.0 μg/L, 26.68–151.78), and in the same way, between the group that consumed iodized salt (153.65 μg/L, 85.55–185.93) and the group that did not use it regularly (46.15 μg/L, 28.28–89.23). We also found differences in TSH levels according to the same criteria for consumption of iodized salt and iodized supplements in the first trimester of pregnancy.

No correlation was found between TSH and maternal ioduria values in the first trimester of gestation with the weight or length of the newborn. Nor was there a correlation with the gestation week at the time of delivery. However, we found statistically significant differences between the APGAR scores of children whose mothers had an iodine sufficiency level in the first trimester compared to the children of mothers with iodine deficiency. Similarly, the APGAR scores of children whose mothers had a TSH > 4 have significantly better APGAR scores than the children of mothers with a TSH < 4.

Likewise, we found statistically significant differences between the measurements of the newborns depending on whether their mothers smoked. The children of non-smoking women weighed an average of 189.98 g (95% CI 38.2–341.77) and measured 0.79 cm (95% CI 0.079–1.508) more than the children of smokers (*p* < 0.05). The mean self-reported tobacco use in the first trimester was 6.0 cigarettes per day (SD = 4.4). Dividing the women into two groups, smokers and non-smokers, we found that the anthropometric values of the newborns are higher in both groups in women who took iodized supplements or iodized salt in the first trimester of pregnancy, although these differences are not statistically significant.

Of the total, 48.4% of children of mothers who took an iodized supplement obtained the highest APGAR score at one minute after birth, compared to 19.5% of children of mothers who did not take an iodized supplement in the first trimester of gestation (*p* < 0.05).

**Figure 1:**
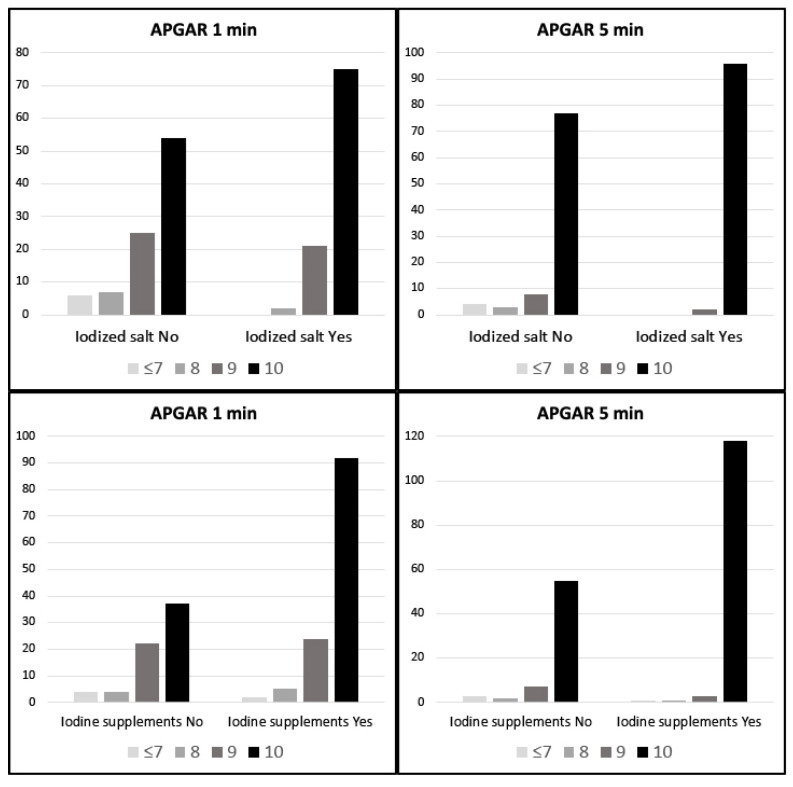
Global APGAR score of newborns according to intake of iodized salt and iodine supplements.

**Figure 2:**
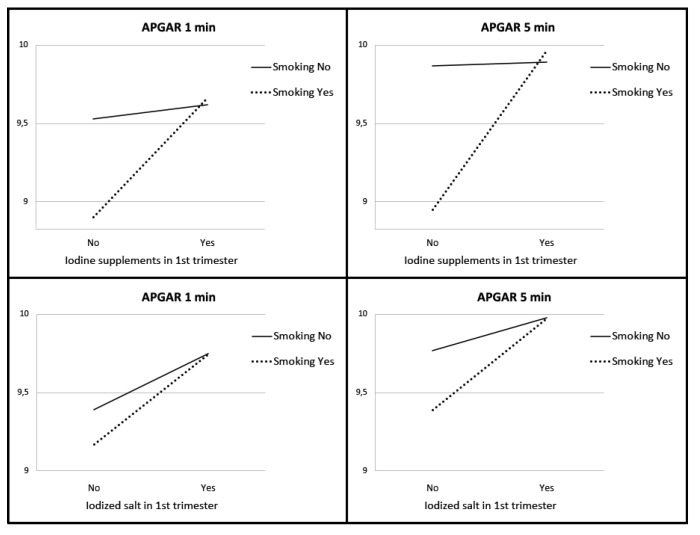
APGAR score of newborns according to smoking, intake of iodized salt, and iodine supplements.

We found statistically significant higher APGAR scores in newborns depending on whether their mothers took iodine supplements or iodized salt before pregnancy and during the first trimester (*p* < 0.05). As can be seen in Figure 1, pregnant women who did not take iodine supplements showed lower APGAR scores for their newborns. The same happened with pregnant women who did not take iodized salt. In the group of smokers, we found significantly higher APGAR values in women who took iodized supplements or iodized salt in the first trimester of pregnancy. In the group of non-smokers, we found these significant differences with respect to the consumption of iodized salt (Figure 2).

## 4. Discussion

Previous studies report that pregnant women with higher TSH levels before and during pregnancy showed an increase in caesarean sections and an increased risk of unfavorable obstetric outcomes such as spontaneous abortions during the first trimester and preterm deliveries [39,40,41] and Maternal TSH levels (>4 mIU/L) were associated with a higher risk of prematurity and neonatal respiratory distress syndrome (NRDS) compared to pregnant women with TSH levels (≤4 mIU/L) [42]. However, they did not find statistically significant results in the association of maternal TSH levels (>4 mIU/L) with an increased risk of loss of fetal well-being, pre-eclampsia and eclampsia, and low birth weight (<2500 g). Furthermore, pregnant women with urinary iodine levels between 150 and 249 μg/L had a lower prevalence of pre-eclampsia, placenta previa, and risk of loss of fetal well-being than the reference group (ioduria < 150 μg/L) [43]. On the other hand, pregnant women with clinical and subclinical hypothyroidism were more likely to have a preterm birth, and clinical hyperthyroidism was significantly associated with a higher rate of miscarriage and a higher risk of loss of fetal well-being. However, in our study we were unable to link TSH levels with a higher prevalence of abortions or obstetric complications (data not shown).

The increase in TSH observed in women taking iodized salt or iodized supplements in the first trimester may be due to a transient altering effect on the thyroid gland, which occurs as a result of the sudden increase in daily iodine intake [44,45,46], so it is possible that we have witnessed the activation of the self-regulatory mechanisms of iodine metabolism in even physiological quantities within the thyroid gland produced by chronic iodine deficiency states [47].

The maternal TSH level in the first trimester of gestation, the anthropometric values (weight and length) of the newborns, and the APGAR test score one or five minutes after birth were compared. We determined that the TSH values were not related to the somatometric parameters of the newborns, coinciding with other studies [48,49] in which they did not observe a significant association in pregnant women with subclinical hypothyroidism and low birth weight, when compared to euthyroid women. The relationship between subclinical hypothyroidism and APGAR scores is unclear. While several studies reported that women with subclinical hypothyroidism had no adverse outcomes in their newborns or found no significant differences with respect to the children of euthyroid women [49,50,51,52,53], other studies found that low APGAR scores were associated with mothers with subclinical hypothyroidism [48,54,55]. Our results, however, show a significant association between higher levels of TSH during the first trimester of gestation with higher scores in the APGAR test. Future studies will be necessary to clarify the influence that subclinical hypothyroidism exerts on the placenta and the fetus during pregnancy and on the fetal capacity to tolerate stress and, therefore, on APGAR scores at birth. Although there is greater evidence of an adequate intake of iodine in pregnancy promoting psychomotor development of the child [3,4,5,6,7,8,9], we found few studies that relate the consumption of iodized salt and iodine supplementation in pregnancy to the anthropometric values of the newborn and the APGAR test one or five minutes after birth.

We did not find an association between maternal TSH levels in the first trimester and gestational age at delivery. Leon et al. [56] observed that maternal TSH during the first half of pregnancy were inversely related to birth weight and a higher risk of small-for-gestational-age (SGA) newborns. Likewise, a meta-analysis [57] found significance between maternal subclinical hypothyroidism with a lower mean birth weight than euthyroidism. They also found that isolated hypothyroxinemia was associated with a lower risk of SGA than euthyroidism and a higher mean weight at birth. As can be seen in previous studies, and despite our results, thyroid alterations during pregnancy could be related to the week of gestation at the time of delivery.

In a cross-sectional study carried out in Brazil [33], in term newborns, birth weight decreased as the number of cigarettes smoked daily increased. The mean birth weight of newborns whose mothers smoked was 320–435 g lower compared to newborns of non-smoking mothers. Likewise, in a meta-analysis [35], maternal smoking was associated with a reduction in fetal biparietal diameter in the second and third trimesters of gestation, and the length of the femur decreased in the second and third trimesters. Higher maternal smoking was associated with lower z-score in head circumference in the second and third trimesters compared to pregnant women who smoked less. Delgado et al. [32] observed a causal relationship between maternal smoking and low birth weight, as well as a reduction in length and other anthropometric measures, causing an overall effect on fetal body composition. However, they found no significant differences between APGAR score values for the children of smoking mothers and non-smoking mothers. These data can be comparable to those found in our study. We found statistically significant differences in weight and height of newborns between the children of smoking and non-smoking mothers, with the children of non-smoking women being larger. Regarding the APGAR scores at one and five minutes, we also did not find significant differences between the children of smoking and non-smoking mothers. However, one of the findings of the present study is that the APGAR score of the newborns of smoking mothers improved significantly if they took iodized salt or iodine supplements in the first trimester of pregnancy.

Currently, there are few studies that link the intake of iodinated supplements or iodized salt and maternal TSH function with the well-being of the newborn. A randomized clinical trial [58] analyzed cognitive results at 5–6 years and obtained no statistically significant differences in the sections of verbal quotient, psychomotor, processing speed, and global scores. In this context, Velasco et al. [59] compared the 300 µg/KI/day supplementation in pregnant women during the first trimester with the anthropometric values, APGAR index, and psychomotor development scales of the newborns. No significant results were obtained between iodine supplementation with anthropometric values and APGAR test scores. However, the newborns did obtain better results on the psychomotor development scales (*p* < 0.05), which evaluate reflexes, control, and motor coordination. Our study found statistically significant differences between mothers who took iodinated supplements and iodized salt in relation to the maximum and optimal score of the APGAR test versus mothers who did not take it during the first trimester.

Our work has some limitations. First, as usual in any observational study, confounding or non-measured factors may compromise the results. On the other hand, the main limitation is not having performed an ultrasound scan to confirm the exact gestational age (it was calculated according to the last menstrual bleed).

## 5. Conclusions

We have not observed that maternal TSH status in first trimester of pregnancy directly influences somatometric values of newborns. However, maternal smoking habits and the consumption of iodized salt and iodinated supplements during the first trimester of pregnancy do affect anthropometric values and neonatal well-being, so that, in addition to insisting on giving up smoking habits, we consider it essential to focus on recommending adequate intake of iodized salt and iodine supplements prior to gestation and at least during the first trimester of pregnancy to achieve better fetal well-being.

## Figures and Tables

**Table 1:** Sociodemographic characteristics of the sample.

	Mean ± SD or *n* (%)	*p*-Value
Maternal age (years)	30.80 ± 5.15	
Advanced Maternal Age (AMA)		
Yes (*n* = 32; 16.8%)	37.97 ± 2.12	0.000 *
No (*n* = 158; 83.2%)	29.35 ± 4.29	
BMI first Trimester (Kg/m^2^)	23.95 ± 4.41	
Gestational age (weeks)	39.34 ± 1.42	
Gestations		
1	80 (42.1%)	
2	64 (33.7%)	
3	33 (17.4%)	
>3	13 (6.8%)	
Gender newborns		
Male	97 (51.05%)	
Female	93 (48.95%)	
Newborn’s weight (g)	3311.70 ± 428.80	
Male	3391.01 ± 440.91	0.009 *
Female	3328.98 ± 401.66	
Newborn’s length (cm)	49.71 ± 2.11	
Male	50.22 ± 2.02	<0.001 *
Female	49.19 ± 2.09	
Type of delivery		
Natural	127 (66.8%)	
Instrumented	42 (22.1%)	
Caesarean section	21 (11.1%)	
Delivery complications		
None	137 (72.1%)	
Loss of fetal well-being	23 (12.1%)	
Prolonged second stage of labor	19 (10%)	
Breech presentation	6 (3.2%)	
Fetal/pelvic disproportion	5 (2.6%)	

* Unpaired Student’s *t*-test.

**Table 2:** Clinical characteristics of the sample.

	n (%)	Mean ± SD	*p*-Value **
Maternal TSH in 1st trimester (mUI/L)	190 (100%)	2.22 ± 1.32	
TSH < 4	160 (84.2%)	1.78 ± 0.87	0.000
TSH > 4	30 (15.8%)	4.61 ± 0.44	
Iodine supplements yes	123 (64.7%)	2.39 ± 1.39	0.013
Iodine supplements no	67 (35.3%)	1.93 ± 1.12	
Iodized salt yes	98 (51.6%)	2.44 ± 1.92	0.021
Iodized salt no	92 (48.4%)	2.00 ± 1.16	
Maternal ioduria in 1st trimester (µg/L) *	190 (100%)	89.15 (41.9–165.15) *	
Ioduria < 150	129 (67.9%)	49.1 (32.1–93.9) *	0.000
Ioduria > 150	61 (32.1%)	184 (166.75–217.75) *	
Iodine supplements yes	123 (64.7%)	50.2(109.7–167.65) *	0.017
Iodine supplements no	67 (35.3%)	49.0 (26.68–151.78) *	
Iodized salt yes	98 (51.6%)	153.65 (85.55–185.93) *	0.000
Iodized salt no	92 (48.4%)	46.15 (28.28–89.23) *	
Newborn’s weight (g)	190 (100%)	3311.7 ± 428.8	
Smokers	70 (36.8%)	3165.5 ± 402.9	0.014
Non-smokers	120 (63.2%)	3355.5 ± 410.3	
Non-smokers			
Iodine supplements yes	73 (60.8%)	3400.59 ± 429.69	0.134
Iodine supplements no	47 (39.2%)	3255.53 ± 371.87	
Iodized salt yes	64 (53.3%)	3388.36 ± 417.43	0.351
Iodized salt no	56 (46.7%)	3318.00 ± 402.33	
Smokers			
Iodine supplements yes	50 (71.4%)	3249.80 ± 447.37	0.702
Iodine supplements no	20 (28.6%)	3203.50 ± 473.25	
Iodized salt yes	34 (48.6%)	3279.58 ± 537.01	0.417
Iodized salt no	36 (51.4%)	3191.00 ± 347.12	
Newborn’s length (cm)	190 (100%)	49.71 ± 2.11	
Smokers	70 (36.8%)	49.1 ± 1.81	0.030
Non-smokers	120 (63.2%)	49.9 ± 1.96	
Non-smokers			
Iodine supplements yes	73 (60.8%)	49.97 ± 2.00	0.576
Iodine supplements no	47 (39.2%)	49.77 ± 1.89	
Iodized salt yes	64 (53.3%)	49.90 ± 2.00	0.961
Iodized salt no	56 (46.7%)	49.88 ± 1.92	
Smokers			
Iodine supplements yes	50 (71.4%)	49.46 ± 2.13	0.784
Iodine supplements no	20 (28.6%)	49.29 ± 2.84	
Iodized salt yes	34 (48.6%)	49.5 ±2.93	0.735
Iodized salt no	36 (51.4%)	49.31 ± 1.50	
APGAR 1 min	190 (100%)	9.53 ± 0.91	
Maternal ioduria < 150	129 (67.9%)	9.33 ± 1.03	0.000
Maternal ioduria > 150	61 (32.1%)	9.95 ± 0.22	
Maternal TSH < 4	160 (84.2%)	9.45 ± 0.96	0.000
Maternal TSH > 4	30 (15.8%)	9.97 ± 0.18	
Iodine supplements yes	123 (64.7%)	9.63 ± 0.87	0.034
Iodine supplements no	67 (35.3%)	9.34 ± 0.95	
Iodized salt yes	98 (51.6%)	9.74 ± 0.48	0.001
Iodized salt no	92 (48.4%)	9.30 ± 1.17	
Non-smokers			
Iodine supplements yes	73 (60.8%)	9.62 ± 1.02	0.615
Iodine supplements no	47 (39.2%)	9.53 ± 0.65	
Iodized salt yes	64 (53.3%)	9.75 ± 0.47	0.028
Iodized salt no	56 (46.7%)	9.39 ± 1.19	
Smokers			
Iodine supplements yes	50 (71.4%)	9.66 ± 0.59	0.001
Iodine supplements no	20 (28.6%)	8.90 ± 1.33	
Iodized salt yes	34 (48.6%)	9.74 ± 0.51	0.009
Iodized salt no	36 (51.4%)	9.17 ± 1.13	
APGAR 5 min	190 (100%)	9.81 ± 0.91	
Maternal ioduria < 150	129 (67.9%)	9.71 ± 1.1	0.004
Maternal ioduria > 150	61 (32.1%)	10.0 ± 0.0	
Maternal TSH < 4	160 (84.2%)	9.77 ± 0.99	0.004
Maternal TSH > 4	30 (15.8%)	10.0 ± 0.0	
Iodine supplements yes	123 (64.7%)	9.92 ± 0.51	0.020
Iodine supplements no	67 (35.3%)	9.60 ± 1.36	
Iodized salt yes	98 (51.6%)	9.98 ± 0.14	0.006
Iodized salt no	92 (48.4%)	9.62 ± 1.28	
Non-smokers			
Iodine supplements yes	73 (60.8%)	9.89 ± 0.64	0.858
Iodine supplements no	47 (39.2%)	9.87 ± 0.34	
Iodized salt yes	64 (53.3%)	9.98 ± 0.13	0.027
Iodized salt no	56 (46.7%)	9.77 ± 0.76	
Smokers			
Iodine supplements yes	50 (71.4%)	9.96 ± 0.20	0.003
Iodine supplements no	20 (28.6%)	8.95 ± 2.35	
Iodized salt yes	34 (48.6%)	9.97 ± 0.17	0.066
Iodized salt no	36 (51.4%)	9.39 ± 1.81	

* Ioduria values are expressed as median (interquartile range) ** Unpaired Student’s *t*-test.

## Data Availability

The datasets used in this study are available from the corresponding author.
